# HTLV-1 persistence and leukemogenesis: A game of hide-and-seek with the host immune system

**DOI:** 10.3389/fimmu.2022.991928

**Published:** 2022-10-10

**Authors:** Benjy J. Y. Tan, Kenji Sugata, Masahiro Ono, Yorifumi Satou

**Affiliations:** ^1^ Division of Genomics and Transcriptomics, Joint Research Center for Human Retrovirus Infection, Kumamoto University, Kumamoto, Japan; ^2^ Department of Life Sciences, Imperial College London, London, United Kingdom

**Keywords:** human T-cell leukemia virus type 1 (HTLV-1), adult T cell leukemia/lymphoma (ATL), viral immune response, cancer immune response, immune escape, clonal persistence, HLA-II

## Abstract

Human T-cell leukemia virus type 1 (HTLV-1), a retrovirus which mainly infects CD4^+^ T cells and causes adult T-cell leukemia/lymphoma (ATL), is primarily transmitted *via* direct cell-to-cell transmission. This feature generates a wide variety of infected clones in hosts, which are maintained *via* clonal proliferation, resulting in the persistence and survival of the virus. The maintenance of the pool of infected cells is achieved by sculpting the immunophenotype of infected cells and modulating host immune responses to avoid immune surveillance. Here, we review the processes undertaken by HTLV-1 to modulate and subvert host immune responses which contributes to viral persistence and development of ATL.

## Introduction

**Figure 1:**
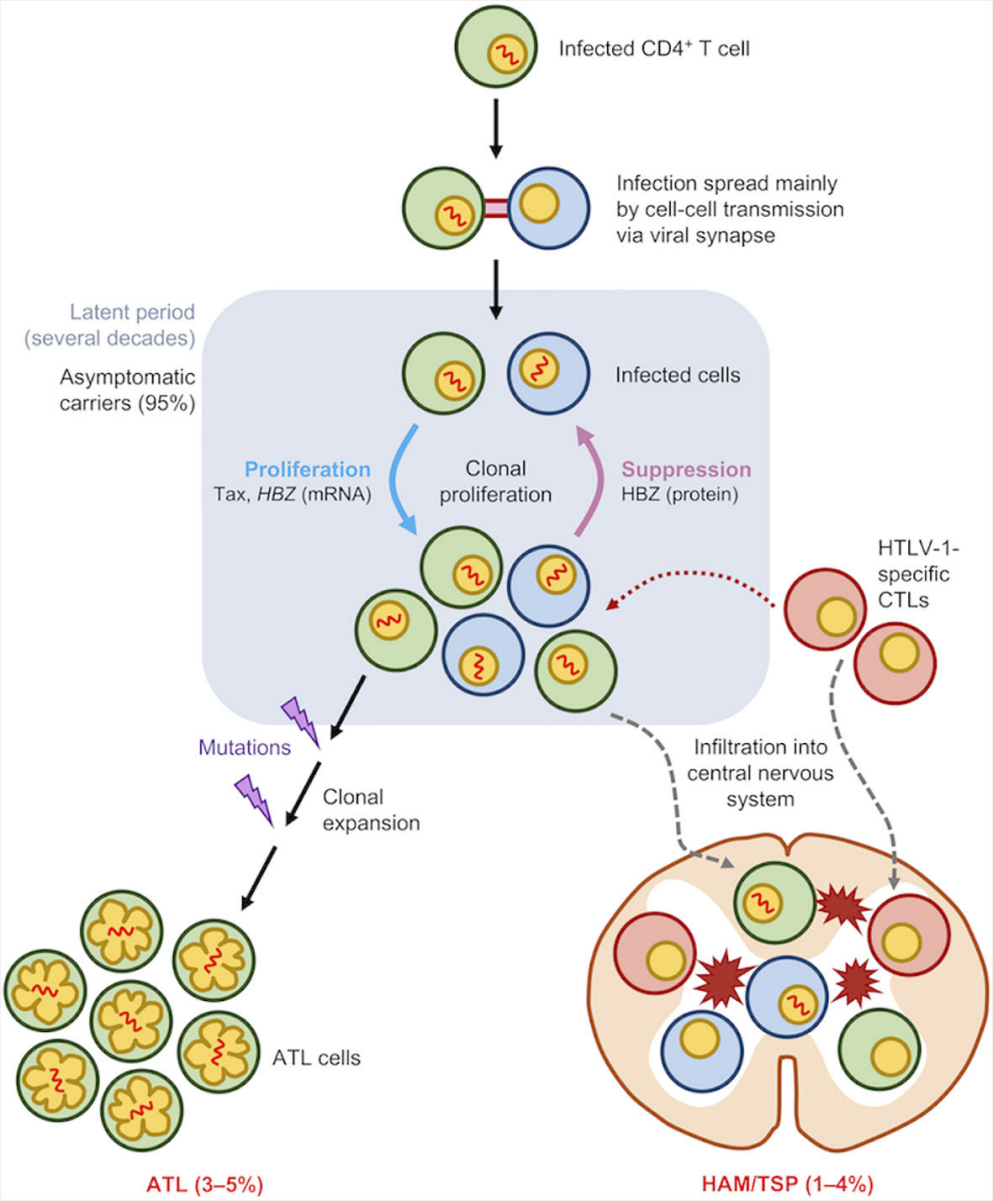
Natural history of HTLV-1 infection. HTLV-1 primarily infects CD4+ T cells and spread mainly by cell-to-cell transmission *via* viral synapse. The pool of infected cells are maintained by clonal proliferation which is promoted by Tax, HBZ mRNA and other viral accessory proteins while HBZ protein and host CTLs act to suppress them. Most HTLV-1-infected individuals remain life-long asymptomatic carriers. However, in approximately 5% of infected individuals, acquisition and accumulation of certain mutations leads to malignant transformation of infected cells into adult T-cell leukemia (ATL) cells. Additionally, about 4% of infected individuals develop HTLV-1-associated myelopathy/tropical spastic paraparesis (HAM/TSP), which is caused by infiltration of infected cells and CTLs into the central nervous system.

Human T-cell leukemia virus type 1 (HTLV-1), the first human retrovirus discovered (1–3), was identified as the etiological agent of adult T cell leukemia/lymphoma (ATL) (4, 5) and to date, one of the only seven human viruses with strong epidemiological links to human cancers (6). The virus primarily infects CD4^+^ T cells and induces a lifelong infection in infected individuals as asymptomatic carriers (ACs) (**Figure 1**). Nevertheless, approximately 3-5% of infected individuals eventually develop ATL, a malignant CD4^+^ T cell neoplasm (7) (**Figure 1**). HTLV-1 is also associated with various other inflammatory diseases including uveitis, dermatitis, arthropathy (8) and most notably HTLV-1-associated myelopathy/tropical spastic paraparesis (HAM/TSP) which affects 1-4% of infected individuals (7, 9, 10) (**Figure 1**).

HTLV-1 is mainly transmitted through three routes: (1) sexual intercourse, (2) breastfeeding, and (3) blood transfusion and needle sharing (11). The peculiar thing about HTLV-1 transmission is that cell-free infection of HTLV-1 is extremely inefficient (12). Instead, HTLV-1 primarily relies on direct cell-to-cell transmission *via* virological synapse (13), viral biofilm (14) or cellular conduits such as tunneling nanotubes (15). Within an infected individual, HTLV-1 presence is then maintained largely *via* mitotic division of infected cells (16) (**Figure 1**) with a minor contribution by infectious spread (17).

In fact, immune cells are constantly surveilling the body to look for and eliminate foreign pathogens or pre-cancerous and cancerous cells (18). This process is called immune surveillance (18) and is carried out by both the innate and acquired immune system. Immune response to HTLV-1 has been extensively reviewed elsewhere (19–22) with most of the recent work focusing on the innate immune responses, including retroviral restriction factors (23, 24) and chemokines (25). HTLV-1 mainly infects CD4^+^ T cells, which are one of the major players of the cell-mediated immune response primarily carried out by T cells (26). Cell-mediated immune response is activated upon antigen presentation by professional antigen-presenting cells (APCs) such as dendritic cells (DCs) and B cells to T cells (26). Elimination of the foreign pathogen or cancerous cells is then carried out through the production of inflammatory cytokines and the killing action of effector cells including cytotoxic T lymphocytes (CTLs), macrophages and natural killer (NK) cells (26). To ensure long-term survival and circumvent this immune surveillance, HTLV-1 would have developed certain traits to modulate host cell’s immunophenotype, immune response and environment so that the infected cells have a survival advantage.

In this review, we will discuss on what is known so far on the virus–host interplay which renders infected cells invisible from immune surveillance and thus contributing to HTLV-1 persistence in infected individuals. The article is divided into 3 major sections which chiefly describes: (1) immune escape mechanisms utilized by HTLV-1-infected cells for persistence, (2) malignant transformation of infected cells plus additional mechanisms employed by leukemic cells, and (3) new findings on the hijack of physiological T-cell activation mechanisms for immune evasion.

## Immune escape mechanisms used by HTLV-1-infected cells for persistence

During the infectious phase, thousands of infected-cell clones are established before plateauing and only those which escaped initial CTL detection continue to live on for decades in infected individuals (27, 28). Here, we will review some of the host and virus factors which sculps the clonal landscape of HTLV-1-infected cells.

### Regulation of HTLV-1 provirus transcription

The HTLV-1 provirus is around 9 kb in length and encodes several structural (*gag*, *pol* and *env*), regulatory (*tax* and *rex)* and accessory proteins [*p12*, *p13*, *p30* and *HTLV-1 bZIP factor (HBZ)*]. Among them, Tax and HBZ are important for the maintenance and proliferation of the pool of infected cells. Tax, which is encoded in the sense strand, is a highly immunogenic protein; while HBZ, encoded in the anti-sense strand, has a lower immunogenicity (29, 30). As such, there is a need for different expression pattern between *tax* and *hbz* to minimize the activity of Tax-specific CTLs.

Transcription from the sense and anti-sense strand of the provirus is controlled by different mechanisms. Sense strand transcription is strongly enhanced by Tax protein, which recruits cAMP response element binding protein (CREB) and the transcriptional coactivators CBP/p300 to the Tax response elements in the 5’ long terminal repeat (LTR) (31). This transcriptional burst is then suppressed by the viral proteins p30, which binds and retains Tax mRNA in the nucleus (32), and HBZ, which interacts with the KIX domain of p300 (33). Interestingly, HBZ acts in a negative feedback loop to control provirus expression and re-establish provirus latency as a small surge of HBZ transcription is observed during the late stage of the Tax burst (34).

Epigenetically, the 5’ LTR is heavily methylated which silences sense strand transcription. However, this only extends up to roughly three quarters of the provirus with the HBZ and 3’ LTR remaining free of DNA methylation, allowing continued expression of HBZ (35, 36). This unique pattern of epigenetic modification is achieved through the insulator-binding protein CCCTC-binding factor (CTCF) that acts to define boundaries between transcriptionally active and inactive regions of the genome by restricting the spread of epigenetic modifications (37). Binding of CTCF to the proviral DNA at a defined epigenetic border helps to regulate and modify provirus transcription. Additionally, a novel enhancer region was recently identified near the 3’ LTR which acts to enhance transcription from the 3’ LTR as well as acting as another barrier to prevent spreading of epigenetic modifications towards the 3’ LTR (38).

**Figure 2:**
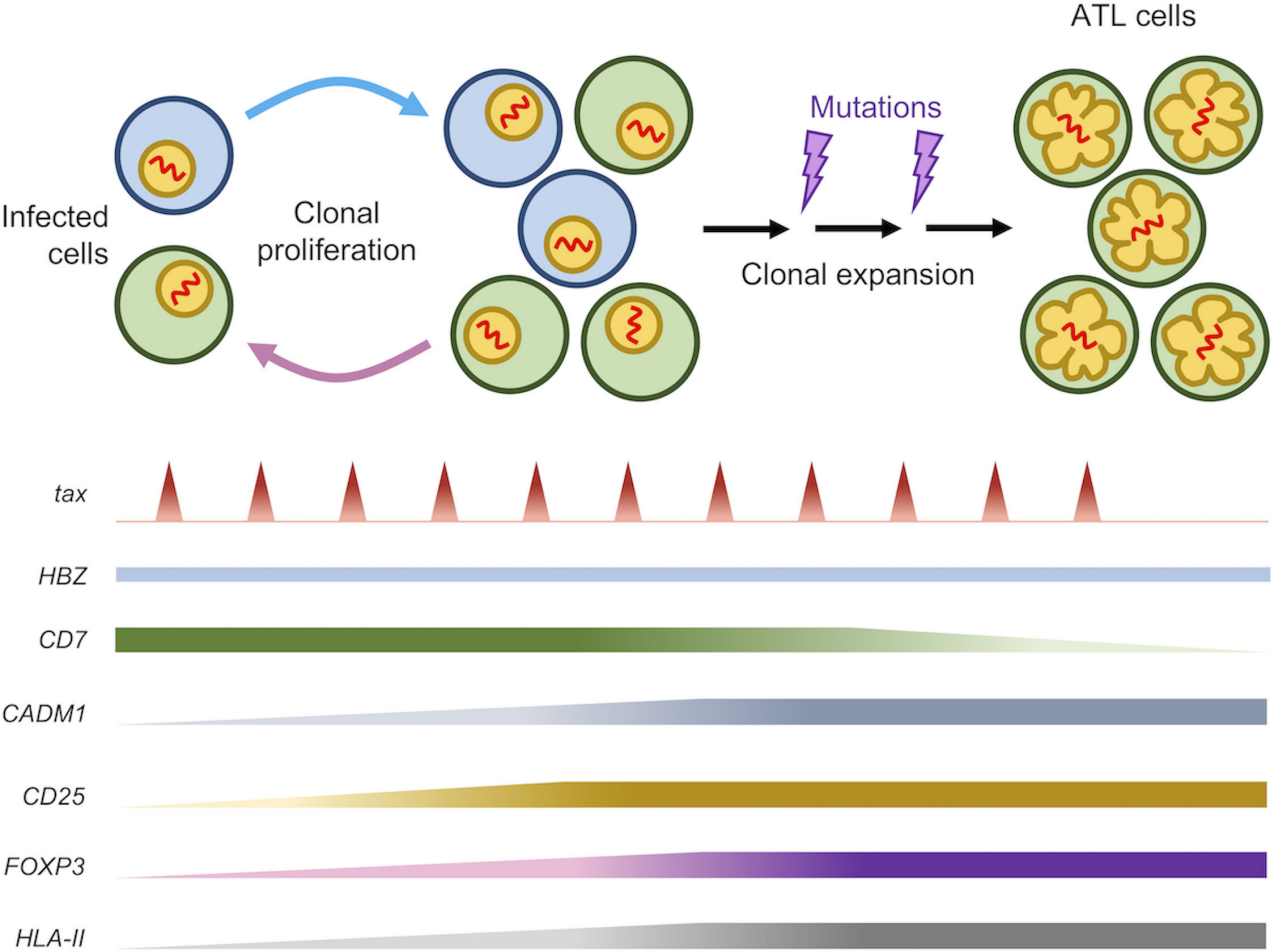
Expression pattern of several related genes during HTLV-1 persistence and leukemogenesis. After a long latency period, about 5% of HTLV-1-infected individuals develop ATL. Both Tax and HBZ are critical for leukemogenesis with the *HBZ* gene being constantly expressed while *tax* is transcribed in rare, short, self-limiting bursts. Infected cells exhibit increased expression of genes related to T-cell activation (*CD25*) as well as Treg (*FOXP3*). Subsequent accumulation of certain mutations during the lifetime of infected clone potentiates leukemic transformation which is accompanied by a loss of CD7 expression and an increase in CADM1 expression. Expression of CD25 and FOXP3, as well as HLA-II, is maintained throughout the latency phase as well as in ATL cells after malignant transformation.

Differences in promoter activity, epigenetic modifications as well as regulatory elements as described above contribute to the contrasting transcriptional kinetics of Tax and HBZ whereby the *tax* gene is being expressed transiently in rare, self-limiting bursts (34, 39) while the *hbz* gene is continuously expressed at low levels (40, 41) (**Figure 2**).

### CTLs response against HTLV-1-infected cells

Among the various viral proteins, Tax is the major viral antigen targeted by CTL (42, 43). The antigen specificity and quality of the CTL response is determined by the human leukocyte antigen (HLA) class I alleles of infected individuals. Several studies reported that individuals with HLA-A*02 had stronger affinities towards various Tax epitopes, especially Tax_11-19_, which confers a lower proviral load due to the selective pressure against Tax-expressing cells. The same studies also revealed that individuals with HLA-Cw*08 are associated with lower proviral load while HLA-B*54 is associated with higher proviral load (44–48).

**Figure 3:**
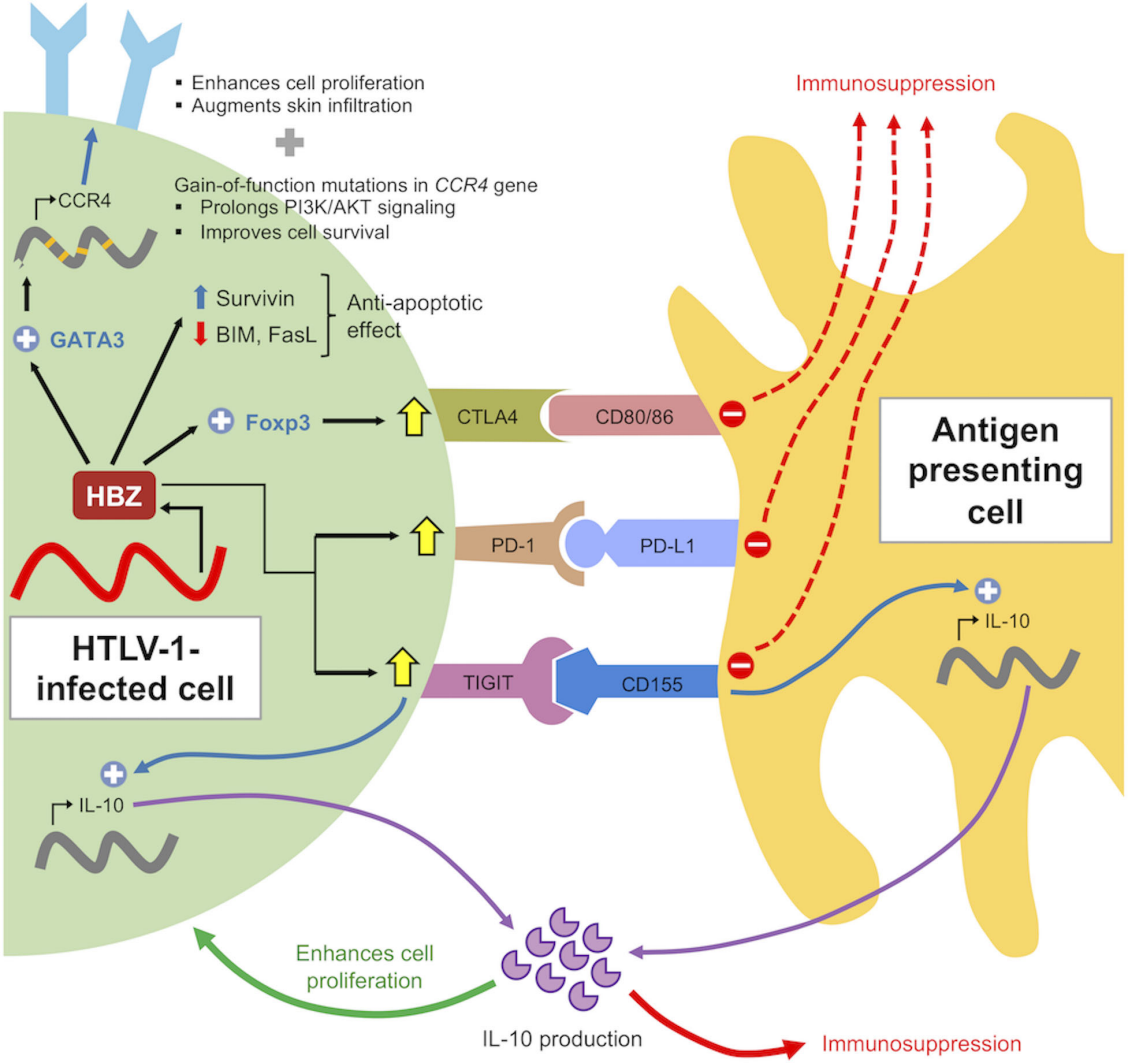
Role of HBZ in immune evasion of infected cells. HBZ induces expression of co-inhibitory molecules to suppress immune activation and confers anti-apoptotic properties by altering expression pattern of pro- and anti-apoptotic proteins. HBZ also induces IL-10 expression in infected cells and antigen presenting cells through TIGIT. IL-10 acts to suppress host immune response as well as enhancing cellular proliferation of infected cells through modulation of STAT signaling by HBZ (not shown).

Notably, HBZ-specific CTLs have a significantly lower frequency than Tax-specific CTLs in infected individuals (30, 49) and are unable to lyse infected cells (29). It has been suggested that the lower frequency and weak immune response of HBZ-specific CTLs is a consequence of the low expression and antigenicity as an immunogen. This is not surprising, given the indispensable roles of HBZ in maintaining the survival and proliferation of infected cells (50, 51) *in vivo* which necessitates the need for persistent expression (**Figure 3**). Such mechanism is not specific to HTLV-1 as it was observed in other oncogenic viruses as well, such as EBNA-1 in Epstein-Barr virus (EBV) (52) and E7 in human papilloma virus (HPV) (53). Furthermore, HBZ in its RNA form elicits additional functions including anti-apoptotic activity and suppression of Tax transcription to evade Tax-specific CTLs (54) (**Figure 3**).

In terms of clonal selection during viral persistence, the expression of Tax confers a disadvantage towards the survival of infected cells whereas HBZ might not. This selective advantage is not specific for ATL cells, but rather a general aspect of HTLV-1 infection, which was demonstrated in a comprehensive DNA-capture-seq analysis whereby in all HTLV-1-infected individuals, the proportion of 5’ defective provirus is around 15% but 3’ defective ones are extremely rare (~2.5%) (55). Analysis of viral genes expression also showed that Tax is not expressed in approximately 60% of ACs and ATL cases but HBZ expression can be seen in all of them (40, 56, 57), which is consistent with the notion that Tax expression is unfavorable for survival *in vivo*.

### Integration preference and provirus structure

HTLV-1 prefers to integrate into transcriptionally active, accessible regions in the host genome with a slight bias to transcriptional start sites (TSS). However, as these proviruses are transcriptionally active, viral genes are expressed resulting in a majority of these cells being eliminated. Most of the infected cells that survived this initial selection are those with integration sites in transcriptionally quiescent regions (58–60). Nevertheless, some of these clones with provirus integrated in transcriptionally active regions do survive and remain in circulation at least partially due to the spatial organization of the chromosome harboring the integrated provirus (61). Although HTLV-1 shows no preference for any given chromosome during initial integration, the clones that survived and persisted *in vivo* are frequently found in the long arm of acrocentric chromosomes (chromosomes 13, 14, 15, 21 and 22) and close to the centromere (61, 62). The centromere-proximal regions of these chromosomes are situated in transcriptionally repressive regions in the nucleus – the nucleolar periphery or nuclear lamina – hence these regions are transcriptionally quiescent which favors the survival of these clones (61).

Besides integration site preference, the proviral structure also determines clonal survival and longevity. Defective proviruses are preferentially selected as these may lack the 5’ LTR and flanking regions coding immunogenic proteins, Tax in particular, which confers survival advantage by evading Tax-specific CTLs (63–65). The loss of the 5’ LTR has been well characterized in ATL patients where there are about 30% defective proviruses (55, 66, 67) while in ACs and HAM/TSP patients, about 15–20% of provirus are defective (55), suggesting that deletion of certain regions from the provirus genome for immune evasion is a general feature of HTLV-1-infected clones (55). Several studies have demonstrated that oncogenesis can still occur despite the absence of Tax, indicating that Tax is important for *de novo* infection but may be dispensable for leukemic transformation and maintenance of the clonal population of infected cells (64). Thus, both proviral integration site and structure plays a role in determining the fate of infected cells *in vivo*.

### Immunophenotype remodeling through Foxp3 expression

Tregs, defined as CD4^+^Foxp3^+^ T cells, play a role in suppressing excessive immune activity and function as ‘guardians’ of immune homeostasis. Interestingly, however, 60–70% of ATL cells express Foxp3 (68–71). Hence, ATL was previously considered as a tumor of HTLV-1-infected Tregs (72). However, recent findings have demonstrated the immunophenotypic modifying properties of HBZ induces some features of Treg in conventional CD4^+^ T cells, although it does not confer suppressive function. HBZ induces Foxp3 expression *via* Smad3-dependent TGF-β signaling (73). However, HBZ then interferes with the DNA binding activity and function of Foxp3 by direct physical interaction (74). Thus, Foxp3^+^ HTLV-1-infected and Foxp3^+^ ATL cells may not always possess the suppressive function of Tregs (75–77). The key question here is why does HTLV-1 alter the immunophenotype of its host cell to mimic the ‘guardians’ of immune homeostasis? One plausible explanation is that Foxp3^+^ infected T cells can evade immune surveillance *via* the expression of immune checkpoint molecules such as CTLA-4, PD-1 and TIGIT and immunoregulatory cytokines including IL-35 and IL-10. The similarities and differences of immunosuppressive mechanism between Treg and HTLV-1-infected cells still require further understanding and further studies utilizing single-cell methods for precise immunophenotyping will provide more evidence on how HTLV-1 evade host immunity (78, 79).

### Host restriction factors

Restriction factors, the first line of antiviral defense, are host cellular proteins that recognize and interfere with specific steps of the virus replication cycle, thus blocking infection. Restriction factors were first described as counteractors of HIV-1 infection and some well-characterized ones include APOBEC3G (apolipoprotein B mRNA-editing enzyme, catalytic subunit-like 3G), SAMHD1 (sterile alpha motif and histidine aspartate domain-containing protein 1), Tetherin/BST-2 (bone marrow stromal antigen 2) and TRIM5α (tripartite motif 5α) (80).

However, as HTLV-1 rarely produce free viral particles and spread primarily *via* cell-to-cell transmission, CTL response against immunogenic viral proteins, particularly Tax, is the predominant immune response against HTLV-1, especially during the initial stages of infection with the role of restriction factors remain poorly understood (23, 81). Nevertheless, in view of the complexity of clonal persistence and the wide spectrum of associated diseases caused by HTLV-1, mechanisms of intrinsic immunity may yet have a role to play. Studies pertaining to HTLV-1 restriction factors are rather limited with the majority of them discovered through studies in HIV-1 infection. The role of restriction factors in HTLV-1 infection has been extensively reviewed elsewhere (23, 24) and is summarized briefly in **Table 1**.

**Table 1:** Restriction factors and their impact on anti-HTLV-1 immunity.

Restriction factor	Impact on HTLV-1
APOBEC3G (A3G)	Can be incorporated into HTLV-1 particles but is weakly susceptible to A3G activity and limited by a peptide motif in the nucleocapsid (82, 83) Generates nonsense mutations *in vivo* but as it targets the minus strand during reverse transcription, the *HBZ* gene is spared which in part explains the constant expression of HBZ and sense-strand silencing (84)
BST2 (Tetherin)	Minimal effect due to efficient spread of HTLV-1 virions *via* virological synapse (85)
SAMHD1	Aborts HTLV-1 infection of myeloid lineage cells *via* STING-mediated apoptosis (86)
TRIM5α	Little information available; but an association is observed between TRIM5α polymorphisms with proviral load (87, 88)
miR-28-3p	MicroRNA which targets a sequence within the HTLV-1 gag/pol mRNA, reducing viral replication and gene expression (89)

## Malignant transformation of infected cells and additional mechanisms employed by leukemic cells

Among the many infected clones, only a selected cell clone will eventually undergo leukemic transformation. The long latent period before ATL onset suggests that malignant transformation occurs when HTLV-1-infected cells acquire a certain set of genetic and epigenetic alterations (90–92) while escaping host’s immune surveillance. Once HTLV-1-infected cells become malignant, they will have to escape not only HTLV-1-specific immune surveillance but also cancer-specific ones (93).

### Modulation of immune checkpoint expression

HTLV-1 is also known to modulate the expression of immune checkpoint molecules on the cell surface of infected cells for immune evasion. Several studies have shown that HTLV-1 infection upregulates both the expression of stimulatory and inhibitory immune checkpoint molecules and is further augmented by leukemic transformation and ATL progression (78, 79, 94). For instance, the viral protein Tax is involved in the overexpression of OX40 (95, 96), a costimulatory molecule which promotes cellular proliferation, enhances cell survival and suppresses Treg activity (97). Interestingly, HTLV-1 selectively enhances expression of particular coinhibitory receptors while suppressing the others. For example, Kinosada et al. (98) showed the involvement of the viral protein HBZ in this process whereby HBZ enhances the expression of TIGIT and PD-1 but suppresses the expression of BTLA and LAIR-1 (98).

TIGIT is a competitive inhibitor of the costimulatory receptor CD226 for binding with the CD155 ligand, resulting in poor T cell activation. Physiologically, TIGIT-mediated signaling inhibits T cell proliferation through the interaction of its cytoplasmic ITIM domains with the THEMIS : SHP complex (99). However, HTLV-1-infected and ATL cells can proliferate *in vivo* despite the upregulation of TIGIT, indicating that downstream TIGIT signaling is impaired. This impairment thus enhances cellular proliferation and is mediated by HBZ which interacts with THEMIS and weakens its interaction with the phosphatases SHP1 and SHP2 (100). Additionally, HBZ also suppresses CD226 expression (100).

An increase in TIGIT-mediated signaling was correlated with increased IL-10 production as shown in TIGIT+CD4+ T cells of HBZ-transgenic mice (100). Likewise, an elevated IL-10 levels was observed in the serum of HTLV-1-infected and ATL patients (101, 102). This increased IL-10 production not only occurs from DCs, but from infected T-cells as well (100, 102). IL-10 is an immunoregulatory cytokine with the functions of suppressing inflammation and Th1 responses (103). Therefore, this elevated expression leads to the generation of an immunosuppressive microenvironment which enhances survival of HTLV-1-infected and ATL cells. Furthermore, IL-10 was reported to support proliferation of ATL cells despite not known to promote T cell proliferation normally (104). This stark contrast of IL-10 function is attributed to the viral protein HBZ as it can modulate IL-10 signaling by interacting with STAT3 (105). This indigenous strategy by HTLV-1 enables the virus to modulate IL-10-mediated signaling pathways for suppressing host immune response in a paracrine manner, while supporting proliferation of infected cells in an autocrine manner.

The role of PD-1 upregulation in HTLV-1 infection is much less established. Expression of PD-1, as well as its ligand PD-L1, is shown to be augmented in HTLV-1-infected individuals and ATL patients (106, 107). Several studies showed that upon PD-1/PD-L1 blockade, CTL function was restored indicating operating PD-1/PD-L1 axis during HTLV-1 infection which diminishes CTL function (106–108). The PD-1 ligands, PD-L1 and PD-L2, are expressed on the surface of dendritic cells and reverse signaling from these ligands stimulates IL-10 expression and confer dendritic cells with an immunosuppressive phenotype (109). Thus, PD-1 upregulation by HTLV-1 in infected cells is a possible mechanism for escaping immune surveillance. Another study by Koya et al. (78) reported that upregulated PD-L1 in malignant cells can be transferred to the microenvironment and alter the anti-tumor immune response (78). However, contrary to the reports above, Takeuchi et al. (110) reported a significant association between PD-L1 expression and improved survival of ATL patients (110). Additionally, similar to TIGIT, the suppressive signaling from PD-1 is impaired *via* HBZ interaction with the THEMIS : SHP complex, thus avoiding growth suppression (98, 100). Furthermore, a clinical trial using the anti-PD-1 antibody, nivolumab, showed that all three participants showed dramatic progression of ATL with a rapid expansion of predominant ATL clones, which was a result of an unanticipated loss of ATL suppression rather than a selective advantage for a specific clone after PD-1 blockade (111, 112). These contrasting findings indicate that further studies are warranted to understand the role and importance of PD-1/PD-L1 upregulation by HTLV-1 and whether PD-1/PD-L1 blockade can be used efficiently as immunotherapy for ATL patients.

### CCR4 expression

CCR4 expression is a well-known hallmark of ATL cells (113). CCR4 upregulation is reported to be induced by HBZ-mediated GATA3 expression which then activates the promoter of the *CCR4* gene. Upregulated CCR4 is associated with enhanced cellular proliferation and chemotactic properties of ATL cells (114). Additionally, HTLV-1-infected cells produces IFN-γ (115), which induces production of the CCR4 ligands CCL17 and CCL22 in keratinocytes and endothelial cells. This chemoattracts HTLV-1-infected cells and augments infiltration into the skin (114). CCL22 production in infected cells is also enhanced by the Tax protein, which in turn chemoattracts and maintains a high frequency of CD4^+^Foxp3^+^CCR4^+^ regulatory T cells (Treg) in circulation. These Treg in turn suppresses and reduced the efficiency of HTLV-1-specific CTL response which promotes survival of ATL cells (70, 116). Furthermore, frequent gain-of-function mutations are observed in the *CCR4* gene in ATL patients. This improves cellular metabolism and survival by prolonging the PI3K/AKT signaling (90, 117). These findings spurred the way for the development of mogamulizumab, a monoclonal antibody against CCR4, which was currently being adopted for treatment of relapsed ATL (118–121).

## New findings on the hijack of physiological T-cell activation mechanisms for immune evasion

We have thus far discussed on the immunomodulation mechanisms of HTLV-1 and the role of HBZ which is summarized in **Figure 3**. It is well established that HTLV-1-infected and ATL cells exhibit a highly activated phenotype. Physiologically, activated T cells are not long-lived and will undergo apoptosis to restore T cell homeostasis, which is largely achieved by cytokine deprivation and negative regulators such as Foxp3 and CTLA4 (122). HTLV-1-infected and ATL cells, which are highly activated, also exhibits a sustained expression of Foxp3 and CTLA4 (**Figure 2**) but these cells are not targeted for cell killing. This implies that HTLV-1 must have developed ways to avoid these negative regulatory mechanisms while maintaining a highly activated phenotype.

HLA class II (HLA-II) are important molecules for the regulation of immune responses by CD4^+^ T cells and normally found only on professional APCs and in T cells after activation. We have recently shown that HTLV-1-infected cells also upregulate HLA-II to present antigen to T cells. However, HTLV-1-infected cells are not efficient APCs as they lack the CD28 ligands CD80/CD86 and cannot provide the key costimulatory signaling in T cells (79). This in turn may contribute to the immune escape of infected cells as the lack of costimulatory signals make responder T cells anergic, in a manner similar to tolerogenic DCs (123). The suppression of T cell activation by tolerogenic DCs is mediated by IL-10 (see above) and a similar mechanism may be operating here as well. Additionally, our study showed that T cells express anergy-related genes upon stimulation by HLA-II on HTLV-1-infected cells, which suggests that HLA-II induction in HTLV-1-infected cells may induce other mechanisms for T cell anergy to inhibit anti-HTLV-1 T cell response.

Additionally, HTLV-1-infected cells also upregulate the expression of *CIITA*, a master regulator of HLA-II expression. We showed that promoter III of *CIITA* was more open in infected cells and its activity increases proportionally to levels of Tax (79) which suggests that Tax contributes to the upregulation of HLA-II during HTLV-1 infection.

In addition to the role in inducing stable HLA-II complexes, previous studies showed that CIITA is a potent transcriptional repressor for Tax-mediated HTLV-1 expression and a potential host restriction factor (23). Tax-mediated HTLV-1 expression is induced by the assembly of a multiprotein promoter complex containing CREB, CBP and PCAF on the viral LTR promoter (31). Tosi et al. (124) showed that CIITA disrupts the assembly of this promoter complex by physically interacting with Tax, thus inhibiting viral replication (124). Furthermore, it was also reported that CIITA can inhibit Tax-mediated NF-κB activation (125). This implies that CIITA may be crucial to counteract HTLV-1 infection and oncogenic transformation. Therefore, it is unexpected that HTLV-1-infected cells, which rarely express Tax, exhibits a high CIITA activity with increased HLA-II expression.

**Figure 4:**
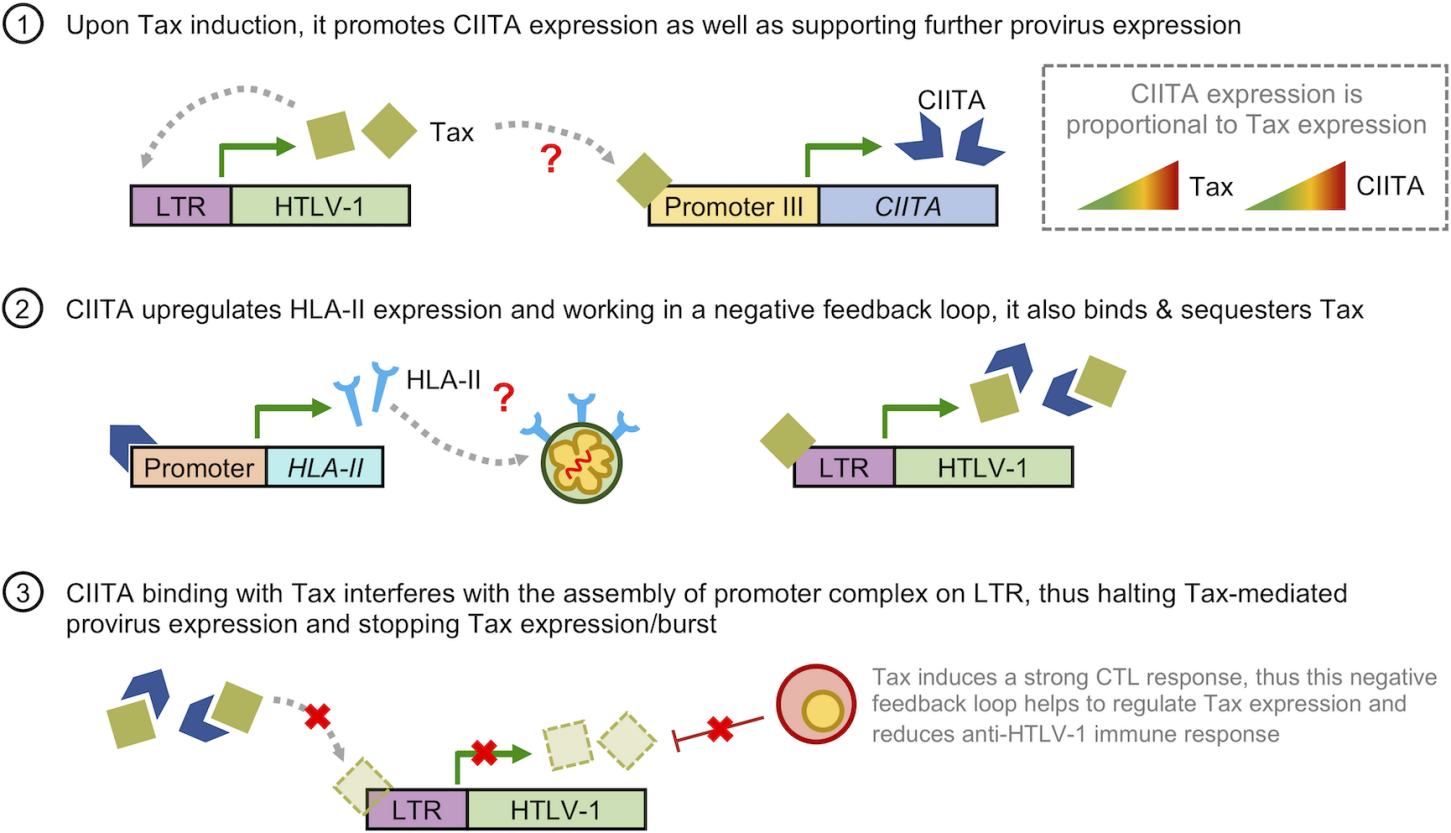
Hypothetical model on how HTLV-1 hijacks the CIITA-HLA-II axis for immune evasion. (1) Upon induction of Tax either during initial infection or a Tax burst, it promotes CIITA expression as well as further supporting HTLV-1 provirus expression in a positive feedback loop. As the level of Tax increases, so does the levels of CIITA. (2) CIITA induces expression of HLA-II-related genes, leading to upregulation of HLA-II molecules on the cell surface of HTLV-1-infected and ATL cells. This confers an immunosuppressive phenotype to these cells. Additionally, CIITA also binds and sequesters Tax. (3) Working in a negative feedback loop, binding of CIITA and Tax reduces the amount of Tax available for the assembly of promoter complex on the LTR, leading to a reduction in Tax-mediated expression of the HTLV-1 provirus, thus halting Tax expression or burst. Additionally, this also reduces the amount of Tax available for antigen presentation to Tax-specific CTLs, thus reducing CTL activity against HTLV-1. The red question marks in (1) and (2) indicates the points in which the regulation of CIITA in HTLV-1 infection is still unclear; namely (1) how Tax interacts with the promoter of CIITA, and (2) how CIITA and HLA-II expression is maintained throughout the latent period.

To reconcile the evidence above, here we propose the model that HTLV-1 hijacks the CIITA-HLA-II axis to enhance its survival and persistence *in vivo* (**Figure 4**). Firstly, irrespective of when Tax expression occurs (either during initial infection or a Tax burst in the latency phase), induction of Tax leads to increased CIITA expression. This then enhances presentation of HLA-II molecules on the cell surface of infected cells which confers infected cells with an immunosuppressive phenotype. CIITA can also bind and sequester Tax to work in a negative feedback loop, thus suppressing Tax-mediated expression. This hijack of host’s cellular factors allows HTLV-1 to halt Tax expression or burst, thus silencing the provirus. Additionally, this also helps to reduce the amount of Tax available for antigen presentation, thus minimizing CTL response against the virus. However, there are still several ambiguous points, chiefly: (1) how Tax interacts with the promoter of CIITA, and (2) how the expression of CIITA and HLA-II is maintained throughout the latent period. We speculated that it is highly possible that similar to CCR4 expression in ATL cells (90, 113, 117), infected cells acquire genetic or epigenetic alterations associated with constitutive expression of the CIITA gene. Another possible explanation is that infected cells modulates expression of certain microRNAs which sustains CIITA expression, similar to how epigenetic downregulation of miR31 expression leads to NFκB activation in the absence of Tax (126). Secondly, by inducing HLA-II expression in host cells, HTLV-1 modulates the immune system to be less responsive and effective against Tax-expressing cells. This dual role hypothesis certainly warrants further investigation to elucidate the role of CIITA in HTLV-1 infection.

## Concluding remarks

HTLV-1 has developed different and unique strategies in order to escape immune surveillance and induce disease in hosts. This remarkable ability of HTLV-1 to outmaneuver host immune surveillance while maintaining a pool of viral reservoir is the major obstacle in drafting effective treatment and prevention strategies. We expect that further uncovering of the immune escape mechanisms of HTLV-1 will lead to the development of innovative methods to reconstitute and restore normal immune homeostasis.

## Author contributions

BT and YS designed the article concept and scope. BJYT wrote the manuscript and conceptualized the figures. KS, MO, and YS provided significant inputs and critically revised the manuscript. All authors contributed to the article and approved the submitted version.

## Funding

This work was supported by JSPS KAKENHI Grant Number JP20240141 and AMED Grant Numbers JP22wm0325015 and JP22jm0210074 to YS.

## Conflict of interest

The authors declare that the research was conducted in the absence of any commercial or financial relationships that could be construed as a potential conflict of interest.

## Publisher’s note

All claims expressed in this article are solely those of the authors and do not necessarily represent those of their affiliated organizations, or those of the publisher, the editors and the reviewers. Any product that may be evaluated in this article, or claim that may be made by its manufacturer, is not guaranteed or endorsed by the publisher.
